# Autophagy Modulators in Coronavirus Diseases: A Double Strike in Viral Burden and Inflammation

**DOI:** 10.3389/fcimb.2022.845368

**Published:** 2022-03-24

**Authors:** Rafael Cardoso Maciel Costa Silva, Jhones Sousa Ribeiro, Gustavo Peixoto Duarte da Silva, Luciana Jesus da Costa, Leonardo Holanda Travassos

**Affiliations:** ^1^ Laboratório de Imunoreceptores e Sinalização Celular, Instituto de Biofísica Carlos Chagas Filho, Universidade Federal do Rio de Janeiro, Rio de Janeiro, Brazil; ^2^ Laboratório de Genética e Imunologia das Infecções Virais, Departamento de Virologia, Instituto de Microbiologia Paulo de Góes, Universidade Federal do Rio de Janeiro, Rio de Janeiro, Brazil

**Keywords:** coronaviral infection, viral replication, inflammation, tissue damage, autophagy

## Abstract

Coronaviruses are the etiologic agents of several diseases. Coronaviruses of critical medical importance are characterized by highly inflammatory pathophysiology, involving severe pulmonary impairment and infection of multiple cell types within the body. Here, we discuss the interplay between coronaviruses and autophagy regarding virus life cycle, cell resistance, and inflammation, highlighting distinct mechanisms by which autophagy restrains inflammatory responses, especially those involved in coronavirus pathogenesis. We also address different autophagy modulators available and the rationale for drug repurposing as an attractive adjunctive therapy. We focused on pharmaceuticals being tested in clinical trials with distinct mechanisms but with autophagy as a common target. These autophagy modulators act in cell resistance to virus infection and immunomodulation, providing a double-strike to prevent or treat severe disease development and death from coronaviruses diseases.

## Introduction

Infectious diseases have been responsible for pandemics and caused billions of deaths during human history. The emergence of antimicrobial therapies and sanitary improvements dramatically contributed to increased life expectancy worldwide (Ventola, 2015). In contrast to antibacterial agents, antiviral pharmaceuticals are radically scarce due to viral dependence on host metabolism, making it more difficult to find highly active and selective compounds. This problem becomes especially critical during significant pandemics such as that caused by severe acute respiratory syndrome coronavirus 2 (SARS-CoV-2), the etiologic agent of COVID-19, originated in Wuhan, Hubei Province, China, which has affected around 350 million people with 5,61 million deaths worldwide.

SARS-CoV-2 is an enveloped positive single-strand RNA virus (ssRNA+) that belongs to the betacoronavirus (βCoV) genus and is related to SARS-CoV and the Middle East respiratory syndrome-coronavirus (MERS-CoV), the causative agents of recent outbreaks in 2003 and 2012, respectively (Zhu et al., 2020). COVID-19 clinical presentations are highly variable, ranging from pauci- or asymptomatic to critical infections characterized by respiratory failure, shock, or multiorgan dysfunction, requiring hospitalization in intensive care units, jeopardizing private and public health systems worldwide.

Although the response to COVID-19 has led to unparalleled research efforts for the development and distribution of vaccines leading to an essential reduction in COVID-19 cases and disease severity, currently, breakthrough infection still occurs amongst fully vaccinated subjects. Thus, urgent and complementary therapeutic strategies to fight the disease are necessary.

Antiviral therapies are mainly targeted to interfere with viral structures. A major drawback frequently encountered is the emergence of drug resistance as a consequence of the selection of mutants due to the inherent viral variability, especially in respiratory viruses (Yan et al., 2014). An alternative to circumvent virus resistance is the design of strategies aiming to regulate host immune responses to infection. This strategy is associated with host protection, reducing the viral burden to promote neutralization, or controlling the detrimental effects of exacerbated inflammation and the resulting tissue damage. As an example, the use of host immune response modulators combined with antiviral agents has been successfully applied, compared to non-combined therapeutic strategies, for the treatment of co-infected patients with hepatitis C virus (HCV) and hepatitis B virus (HBV) (Shih and Liu, 2020). In this case, interferon-alpha (IFNα), an essential cytokine for host resistance, is combined with nucleoside analogs, such as ribavirin and lamivudine (Mavilia and Wu, 2018). Interestingly, SARS-CoV infection also benefits from interferon treatment (Booth et al., 2003). Besides that, corticosteroid treatment of critically ill patients with covid-19 exerts a beneficial effect, controlling inflammatory tissue damage and improving disease tolerance (Sterne et al., 2020).

The modulation of innate immunity, for example after IFNα treatment, can be used against different viruses and could inhibit viral replication, allowing the proper onset of adaptative immune responses. Innate immune recognition of virus initiates several responses to viral molecules such as genomic DNA and RNA or double-stranded RNA (dsRNA) released/formed during viral replication (Wu et al., 2020). These molecules are sensed by pattern-recognition receptors (PRRs) such as Toll-like receptors (TLRs), zinc-finger antiviral protein (ZAP), RIG-I-like receptors (RLRs), and cyclic GMP–AMP synthase- Stimulator of interferon genes (cGAS-STING) pathway to trigger effective antiviral responses, including the production of a myriad of cytokines to induce inflammation and progression of adaptive immune responses (Luo et al., 2020; Wu et al., 2020). In this context, type I interferons (IFN-I), which include IFNα and IFNβ, mediate the induction of both innate and adaptative immune responses through dendritic cells (DCs) maturation promoting increased levels of co-stimulatory molecules, such as the cluster of differentiation 80 (CD80), CD86, and CD40. Mature DCs migrate to draining lymph nodes and present antigens *via* major histocompatibility complex I (MHC-I) to CD8+T cells and MHC-II to CD4+ T cells. CD8+ T cells secrete several pro-inflammatory cytokines and induce cell death of virus-infected host cells, reducing an important source of virus replication. CD8+ T cells induce cell death after recognition of the MHC-I-viral peptide complex expressed by the target cell. CD4+ T cells orchestrate antiviral cellular and humoral immunity. CD4+ T cells, through secretion of distinct cytokines, like IFNγ, increase the microbicidal ability of phagocytes, like macrophages and neutrophils, and promote antibody class-switch and affinity maturation of activated B cells. B cells are the adaptive immunity cells responsible for antibodies secretion, promoting viral neutralization, opsonization, and complement activation (Hoffman et al., 2016).

Regulation of inflammation-mediated tissue damage during viral infections is also an attractive therapeutic target. Inflammatory damage plays an essential role in coronavirus diseases, and anti-inflammatory approaches have been used, especially in the “inflammatory phase” of the infection in which neutralizing antibodies and low extracellular viral loads are found (Desai et al., 2020; Zhang Y. Y. et al., 2020). In these settings, the inflammatory-mediated tissue damage and epithelial cells metaplasia are thought to be the key players in disease progression (Schaefer et al., 2020). Inactivated viruses will not lead to direct cell damage and release of DAMPs (danger-associated molecular patterns). However, it will be responsible for activating several PRRs that are engaged in response to viral pathogen-associated molecular patterns (PAMPs), and, in combination with virus-antibody complexes, it will keep monocytes and neutrophils Fc receptors activated.

## Coronavirus Diseases Pathogenesis

There are several similarities in the pathogenesis of COVID-19 and the diseases caused by SARS-CoV and MERS-CoV, as reviewed elsewhere (Zhang Y. Y. et al., 2020). These viruses cause lower respiratory tract infections and, as such, possess several common symptoms and signs, like non-productive cough (in a small proportion of patients, there is hemoptysis), fever, myalgia, chills, malaise, and shortness of breath. Interestingly, gastrointestinal symptoms can also be present, like vomiting and diarrhea, a feature associated with the diversity of cells infected by these viruses (Zhang Y. Y. et al., 2020), including kidneys and immune cells, like T lymphocytes and macrophages. Thus, lymphopenia can also be a common finding associated with the cytopathic effects of the viruses and a dysregulated immune response. Severe disease caused by these coronaviruses was positively correlated with increased levels of inflammatory cytokines, especially after ten days of symptoms, a phase in which the viral titers are usually decreasing (Lee et al., 2020). A complex interplay between the kinetics and levels of IFN-I and proinflammatory cytokines, such as tumor necrosis factor α (TNFα), interleukin 6 (IL-6), IL-8, IL-1β, and interferon gamma-induced protein 10 (IP-10), seems crucial for resolution or progression to severe disease at this stage (Lau et al., 2013; Zhou et al., 2014; Channappanavar et al., 2016). Thus, severe disease is associated with late high levels of IFN-I, probably a consequence of both viral evasion and interferon antagonism in the early times of infection (Totura and Baric, 2012). In this sense, early IFN-I treatment is associated with MERS-CoV disease protection in mice, and IFN-I therapy was used with promising results for SARS-CoV patients (Booth et al., 2003; Channapanavar et al., 2019), and as we discuss throughout this text, IFN signaling and cellular effects are affected and mediated in part by autophagy. In addition, samples obtained from the lungs of severe MERS-CoV-infected patients presented more than 50 times the levels of IL-1α, IL-1β, and IL-8 than those found in healthy individuals (Alosaimi et al., 2020). Once more, autophagy can be an exciting target in this setting. Autophagy restrains IL-1β release and may contribute to control immunopathology, as this cytokine is also involved in SARS-CoV and SARS-CoV-2 diseases (Rodrigues et al., 2020; Zhang Y. Y. et al., 2020). The kinetics and type of adaptive immune response, including T cell and B cell-mediated immunity, are also crucial for coronavirus diseases’ progression. Severe disease is associated with a more prominent response of the CD4+ T cell subtype Th2 compared to Th1 (Li et al., 2008; Alosaimi et al., 2020; Pavel et al., 2021). Furthermore, increased levels of CD8+ T cells responses were found to be associated with mild disease, highlighting the importance of this cytotoxic T cell population (Zhao et al., 2017; Mallajosyula et al., 2021). Interestingly, hyperactivated CD8+ T cells responses were found in severe patients, indicating that a fine-tuning of the immune responses is crucial for coronavirus disease pathogenesis (Shin et al., 2019; Kang et al., 2020). As we will also explore later in this text, autophagy can influence the activation of both CD4+ and CD8+ T cells (Valečka et al., 2018). Similarly, the levels, kinetics, and type of post-translational modifications in antibody classes are also crucial for coronavirus diseases, and proper regulation will be a central feature for disease progression or control (Hoepel et al., 2021; Zhou et al., 2021). Even though there are many similarities between the diseases caused by SARS-CoV, SARS-CoV-2, and MERS-CoV, some significant differences should be highlighted. MERS-CoV spike (S) protein, which is crucial for coronavirus invasion, binds to the dipeptidyl peptidase 4 (DDP4) receptor (Wang et al., 2013), while S proteins from SARS-CoV and SARS-CoV-2 binds to the angiotensin-converting enzyme 2 (ACE2) receptor (Li et al., 2003; Yang et al., 2020). DDP4 receptor is expressed at higher levels than ACE2 in many different cells such as monocytes and dendritic cells, in which MERS-CoV generates productive infections differently from SARS-CoV (Channappanavar and Perlman, 2017). Epithelial cells from the kidneys, liver, intestines, and prostate also express higher levels of DDP4 compared to ACE2 (van Doremalen et al., 2014). This differential expression between DDP4 and ACE2 might be associated with a higher dissemination rate of MERS-CoV throughout the body and the prevalence of systemic events, like septic shock and multiorgan failure. All these features contribute to the higher mortality of MERS-CoV disease compared to other coronaviruses diseases (Zhang Y. Y. et al., 2020).

## Autophagy: A Contributor to Immune Responses

More recently, it has been demonstrated that the immune system deploys distinct pathways to fight viral infections. One of these strategies is macroautophagy, a crucial stress-induced response (Schmeisser et al., 2014; Caldwell, 2016; Laing et al., 2019). Autophagy is a term derived from the Greek, in which auto means “self” and phagy means “eat.” In cell biology, autophagy describes the ability of cells to degrade its components. Three types of autophagy targets cell components for lysosomal degradation: microautophagy stands for the degradation of macromolecules captured by invaginations or protrusions of the lysosome membrane in a process mediated by the interaction of charged phosphatidylserine in the lysosomal membrane with chaperone heat shock cognate 71KDa protein (Hsc70) (Schuck, 2020); chaperone-mediated autophagy (CMA) stands for a more refined mechanism, in which cargo is not directly sequestered in membranous structures but possesses a five peptide chaperone binding motif that allows its interaction with Hsc70, and consequent interaction with the lysosome receptor lysosomal-associated membrane protein 2A (LAMP2A) (Kaushik and Cuervo, 2018); and macroautophagy, which is a complex multistep process that starts with the formation of the autophagosome, as we will describe later, here referred simply as autophagy.

Autophagy regulates several aspects of host immune response, such as antigen presentation for CD4+ and CD8+ T cells (Valečka et al., 2018); T and B cell development and homeostasis (Arbogast and Gros, 2018); and the secretion of inflammatory mediators (Dai et al., 2017; Tian et al., 2019) and their effects on target cells (Schmeisser et al., 2014; Prieto et al., 2015). For instance, autophagy can restrain intracellular pathways responsible for cytokines secretion, including intracellular PRRs, like RLRs and TLRs, responsible for IFN-I release (Reed et al., 2015; Cotzomi-Ortega et al., 2020). In this context, autophagy negative regulation of mitochondrial reactive oxygen species (ROS) and degradation of macromolecules seems to restrain IFN-I release after RLRs signaling (Tal et al., 2009), while possessing a dichotomic role for TLRs, depending on the circumstances and type of TLR activated (Henault et al., 2012; Zhou et al., 2012; Song et al., 2018). For example, TLR7 endosomal signaling is optimized by viral delivery to endosomes, a process that autophagy seems to contribute (Zhou et al., 2012). On the other hand, acceleration of endosome degradation *via* autophagy can contribute to TLR7-signaling restriction, a feature explored by some viruses like coxsackievirus 16, to circumvent host resistance provided by IFN-I release (Song et al., 2018). Interestingly, autophagy is also induced after IFN-I and lipid mediators (15-epi-lipoxin A4 and resolving D1) signaling as an essential stress response mechanism regulated by inflammatory mediators, leading to viral clearance by xenophagy, in the case of IFN-I, and resolution by 15-epi-lipoxin A4 and resolving D1 (Schmeisser et al., 2014; Prieto et al., 2015; Tian et al., 2019). Thus, different studies demonstrated increased inflammatory conditions in mice with genetic deletions of autophagy-related genes (Reed et al., 2015; Cotzomi-Ortega et al., 2020), reinforcing the importance of autophagy for an appropriate immune response, with an anti-inflammatory role.

## Autophagy: Pathways and Functions

Autophagy was initially described as an adaptive process to starvation, promoting energy maintenance; the recycling of senescent or disabled macromolecules and organelles; and providing the building blocks for *de novo* synthesis of macromolecules. Autophagy is highly conserved among eukaryotes. Several aspects and proteins involved in the autophagy process were first described in yeast, with many orthologs conserved until vertebrates. Nowadays, autophagy functions have been expanded to many distinct aspects of cell biology, such as cell death, signaling regulation, and cell resistance to infections after targeting microorganisms for lysosomal degradation (Bustos et al., 2020).

**Figure 1:**
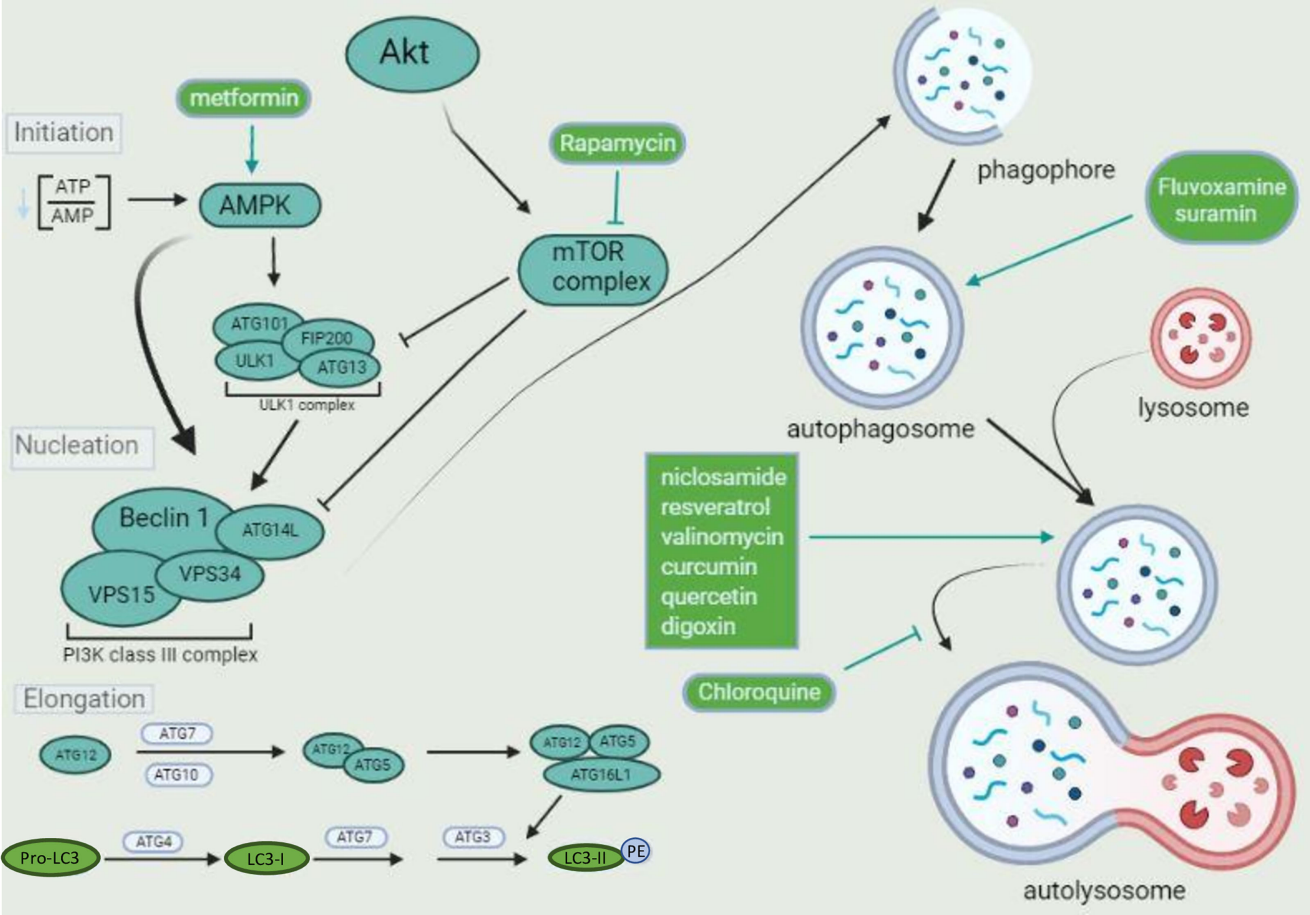
The autophagic flux and its components. Diverse complexes tightly regulate the autophagic pathway. Two kinase complexes are involved in phagophore formation. The ULK1 complex activates the class III PI3K that performs the phospholipid 3-phosphatidyl inositol (PI3) phosphorylation, generating PI3P, which is crucial for phagophore nucleation. Both complexes can be oppositely modulated by distinct kinases, like AMPK, associated with activation, and mTOR complex, associated with inhibition, depending on the amino acid residue targeted for phosphorylation by each kinase. The pharmaceuticals metformin and rapamycin drive autophagosome formation after activating AMPK and inhibition of mTOR complex, respectively. The kinase Akt (or protein kinase B-PKB) is upstream from the mTOR complex, activated by various stimuli. Once a phagophore is formed, it is elongated by different proteins, especially LC3. Pro-LC3 is converted to LC3-I by ATG4. ATG7, ATG3 and the complex formed by ATG12-ATG5-ATG16L1 are crucial for LC3-II formation and binding to the phagophore lipid phosphatidylethanolamine (PE). LC3-II promotes phagophore maturation and closure. The mature autophagosomes are fused with lysosomes and degraded by acid proteases, leading to macromolecules and organelles recycling. Pharmaceuticals that induce lysosome biogenesis, like niclosamide, resveratrol, and valinomycin, promote autophagosomes degradation. Chloroquine is an alkalinizing lysosomotropic agent, inhibiting acid proteases activity and autolysosome degradative function, leading to autophagosome accumulation.

The first step of autophagy is the formation of the autophagosome that, once matured, fuse with lysosomes where acidic hydrolases degrade their components. The formation of the double membrane structure of the autophagosomes (pre-autophagosomal structure-PAS) will require a cluster formed by the proteins Unc-51 like autophagy activating kinase (ULK1), autophagy-related protein 13 (Atg13), focal adhesion kinase family interacting protein of 200 kDa (FIP200), and Atg101 (Lahiri et al., 2019). This complex will phosphorylate and activate several proteins that form another cluster composed of Beclin-1, vacuolar protein sorting 15 (Vps15), Atg14L, and Vps34. This cluster forms the class III PI3K complex that is crucial for autophagosome formation through phosphorylation of the membrane lipid phosphatidylinositol (PI), involved in the recruitment of several adaptor proteins for autophagosome elongation (Lahiri et al., 2019). The elongation of the autophagosome structure is mediated by two ubiquitin-like systems composed by the complex Atg12-Atg5-Atg16L and light chain 3 (LC3) (Lahiri et al., 2019). Atg12 is activated by the protein Atg7 (with a function similar to E1 ubiquitin enzymes) and binds to Atg5 in an Atg10-dependent manner (similar to E2 ubiquitin transferase). The complex Atg12-Atg5 binds Atg16L1 and attaches to the outer membrane of the forming autophagosome, where it recruits the lipidated form of LC3 (LC3-II) (Walczak and Martens, 2013). LC3-II is formed after cleavage of cytosolic LC3 by Atg4, generating LC3-I. LC3-I is activated by Atg7 and transferred to Atg3, which is crucial for binding LC3 to phosphatidylethanolamine (PE) in the autophagosome membrane, forming LC3-II (Tanida et al., 2004). The last step of the autophagy process, the lysosome fusion, is also regulated by another protein complex composed of Beclin-1, Vps34, Vps15, and UV radiation resistance-associated gene protein-UVRAG (Morris et al., 2015). All the proteins in these clusters can be regulated by several other proteins, like Atg9, AMP-activated protein kinase (AMPK), mammalian target of rapamycin complex 1 (mTORC1), E1A Binding Protein P300 (EP300), and others, as described herein another section. In addition, the intracellular trafficking and fusion of autophagosomes with lysosomes are finely coordinated by Rab GTPases and N-ethylmaleimide-sensitive factor attachment receptors (SNAREs) proteins (Ao et al., 2014; Wang et al., 2016). Thus, modulators that affect distinct steps of the autophagic machinery have been described and will also be discussed here (**Figure 1**).

The macromolecules and organelles directed to autophagy must also be tightly regulated. In this sense, autophagy-adaptor proteins act like molecular linkers, allowing interaction between targeted molecules and LC3-II from the autophagosomes for later lysosome degradation. The best-characterized adaptor proteins are p62; nuclear dot protein 52 (Ndp52); histone deacetylase 6 (Hdac6); optineurin (Opt); Neighbor Of BRCA1 Gene 1 Protein (Nbr1); and Tax1 binding protein 1 (Tax1bp1). These adaptors possess multiple domains that allow their interaction with LC3 (LC3 interacting domains-LIR), ubiquitin, and distinct macromolecules, such as the active lipid phosphorylated phosphatidylinositol (PIP) (Johansen and Lamark, 2011).

## The Interplay Between Autophagy and Virus Infections

Viral replication is a major cause of cellular stress, causing the misbalancing of cellular metabolism to produce a considerable number of infectious viral particles (Sanchez and Lagunoff, 2015). Not surprisingly, numerous studies have described an interplay between viruses and the autophagic machinery, with the first report dating from 1965, when the group of George Palade demonstrated the presence of “autolytic vesicles,” later known as autophagosomes, containing poliovirus particles during infection (Carneiro and Travassos, 2016). It is clear that many viruses induce an autophagic response in the infected cell, but the contribution of this pathway to either host antiviral defenses and immune responses or viral replication is variable.

As a piece of fundamental machinery that rapidly responds to diverse types of stress, it is expected that autophagy plays a significant role in viral restriction, through the degradation of viral particles and their components or even host proteins used for viral replication, in a process termed virophagy (Choi et al., 2018). In addition, as previously described here, autophagy impacts host immune responses, which also influences viral replication.

## Inhibition of Autophagy by Viruses

As an essential resistance factor for host cells against some viruses, it is expected autophagy to be inhibited by several viruses in different steps of its pathway to favor virus replication and spread. In agreement with the critical role of mTORC1 in autophagy suppression, some viruses present sophisticated strategies to inhibit autophagy through mTORC1 increased activity. This is the case for Kaposi’s sarcoma-associated herpesvirus (KSHV), a double-stranded DNA virus (dsDNA) that has been shown to induce mTOR activation through its Viral-G protein-coupled receptor (v-GPCR), promoting an important increase in cellular protein synthesis, especially viral ones, and inhibiting autophagy as well (Bhatt and Damania, 2012). Interfering with early steps of autophagy also seems to be a strategy deployed by other viruses such as HIV-1. This is achieved by activating mTOR by the viral envelope protein during entry in dendritic cells, leading to inhibition of immunoamphisomes, which are intermediate/hybrid organelles formed after the fusion of endosomes and autophagosomes. These organelles possess immunostimulatory properties (Blanchet et al., 2010). HIV-1 can also inhibit early steps of autophagy activation by interacting with the viral protein Nef and Beclin-1, resulting in mTORC1 activation, transcription factor EB (TFEB) phosphorylation, and cytosolic sequestration, inhibiting autophagosome maturation (Campbell et al., 2015). Other viruses seem to aim at vesicle nucleation to dampen the autophagic pathway. Human cytomegalovirus (HCMV), a dsDNA virus, encodes TRS1 protein that interacts with Beclin-1 to suppress autophagy (Chaumorcel et al., 2012). Furthermore, Viral B-cell lymphoma 2 (vBCL-2), a viral counterpart of cellular Bcl-2 (cBcl-2), encoded by several viruses directly interacts with Beclin-1, leading to sequestration of this molecule and blunting of autophagy initiation (Yamamoto et al., 1999; Pattingre et al., 2005; Ku et al., 2008). Interestingly, some virus, like Chikungunya virus (CHIKV), a ssRNA+, inhibits mTORC1 after induction of oxidative and endoplasmic reticulum (ER) stresses. In this case, mTORC1 inhibition by CHIKV was associated with increased viral replication and viral protein translation through an eIF4E activation-dependent mechanism, while mTORC1-independent (Joubert et al., 2015). These studies demonstrate that mTORC1 inhibition by CHIKV can promote viral replication, despite a possible induction of autophagy. Thus, in the case of CHIKV, the complex network of virus-host interactions targeting a protein complex (mTORC1) related to different signaling pathways, in the referred case, autophagy and protein translation, will determine the outcome of the infection.

## Autophagy as an Inhibitory Factor for Viral Infections

The interplay of autophagy and CHIKV is complex. A differential role of two autophagy-related adaptor proteins seems crucial for the balance between virophagy and viral replication, with opposite outcomes concerning host cell death. While cellular Ndp52 promoted viral replication, after interaction with the CHIKV nonstructural protein 2 (Nsp2), p62 promoted virophagy and increased survival of the host cells after interaction with viral ubiquitinated capsid. Ndp52 ability to bind Nsp2 is present in permissive human cells but absent in non-permissive cells from mice (Judith et al., 2013). These results highlight critical molecular mechanisms that explain the difference in virus permissivity in two different mammalian cells (Judith et al., 2013). In line with an inhibitory role of autophagy for CHIKV infection, Joubert et al. (2012) showed that autophagy induction inhibits cell death and limits viral propagation. In Sindbis virus infection (SINV), another ssRNA+ virus, Beclin-1 and Atg5 were shown to protect the host against encephalitis (Liang et al., 1998). In the same direction, Smad ubiquitin regulatory factor 1 (SMURF1), an E3-ubiquitin ligase, was demonstrated to drive SINV viral capsid for autophagosomal degradation (Orvedahl et al., 2010). Autophagy-dependent restriction is also observed during Picornavirus (ssRNA+) infection when galectin-8 senses the virus to trigger autophagy and degrade the viral RNA genome (Staring et al., 2017).

It is important to highlight that autophagy-related protein possesses other functions not associated with the autophagic process. Thus, autophagy-independent effects of Atg13 and FIP200 have been described to inhibit viral replication of encephalomyocarditis virus (EMCV), a picornavirus (Mauthe et al., 2016). The authors demonstrated that knockdown of Atg13 and FIP200 in permissive cells, but not other components of the ULK1 complex or autophagy-related proteins like Atg7, increased EMCV replication. Furthermore, no additive effect was demonstrated with the knockdown of both Atg13 and FIP200, suggesting that both proteins are in the same pathway. Though the authors could not determine the exact manner by which Atg13 and FIP200 control viral replication, they did rule out an interplay between IFN mediated virus restriction and these autophagy proteins (Mauthe et al., 2016)

## Autophagy Promotes Antiviral Immune Responses

The protective role of autophagy to host cells can also be achieved independently of virophagy through the induction of immune responses. For example, viral recognition by plasmacytoid dendritic cells relies on autophagy for anti-viral cytokine secretion (Lee et al., 2007), such as IFN-I that hampers viral protein translation and assembly, after inducing interferon-stimulated genes (ISGs), like myxovirus resistance 1 (Mx1) (Verhelst et al., 2012). Curiously, type I interferons also rely on virophagy to exert their antiviral effects (Tian et al., 2019), in a compelling positive feedback loop in the case of TLR7-mediated antiviral signaling. Autophagy also seems to facilitate MHC-II antigen presentation by APCs and activation of CD4+ T cells (Rey-Jurado et al., 2015), and viruses have evolved strategies to inhibit autophagy and restrict recognition by specific CD4+ T cell clones, as demonstrated for Epstein‐Barr virus nuclear antigen 1 (EBNA‐1) (Paludan et al., 2005).

The effector functions of T cells are also influenced by autophagy, likely due to alterations in mitochondrial metabolism (Macian, 2019). At last, autophagy also affects antigen presentation and activation of CD8+ T cells in the context of the MHC-I-peptide complex. Different studies reported conflicting results in this context, showing both positive and negative effects (Van Kaer et al., 2019; Øynebråten, 2020). For instance, autophagy seems to decrease the surface expression of MHC-I, after internalization and degradation. In this regard, genetically deficient DCs for autophagic related proteins (Atg5, Atg7, or Vps34) possess an increased surface expression of MHC-I and viral antigen presentation ability (Mintern et al., 2015; Loi et al., 2016; Parekh et al., 2017). On the other hand, autophagy promotes antigen cross-presentation (Li et al., 2011; Dasari et al., 2016), in which exogenous antigens are presented in the context of MHC-I (Embgenbroich and Burgdorf, 2018). Hence, autophagy induction, after herpes simplex virus 1 (HSV-1) infection, is involved in the increased presentation of viral antigens to CD8+ T cells (English et al., 2009). In conjunction, different studies demonstrate that autophagy interplay with MHC-I peptide antigen presentation is complex, but can be an interesting target to modulate adaptive immunity activation.

## Autophagy as a Promoter of Viral Infection

Autophagy has also been widely reported as a mechanism that promotes some virus replication. The fact that autophagosomes harbor key molecules and provide protection from immune detection makes these compartments a target for creating a replicative niche for many RNA viruses. The formation of double-membrane vesicles (DMVs) has been widely reported during viral infection, which is the case for Picornaviruses. These small RNA viruses use autophagosomes as membrane scaffolds to assemble and replicate their genomic RNA. How these viruses escape virophagy is unclear, and controversial studies about a possible inhibition of autophagosomes and lysosomes fusion do not allow proper conclusions (Kemball et al., 2010; Shi et al., 2016). Picornaviruses have also been shown to use autophagy to induce their non-lytic release. Growing evidence from the literature demonstrates that poliovirus (PV) and coxsackievirus can spread without lysing the cell, through extracellular microvesicles, including autophagosome-derived ones (Bird et al., 2014; Robinson et al., 2014; Granato et al., 2015). In line with a beneficial role for autophagy in PV replication, rapamycin, an inducer of autophagy, strongly up-regulates PV replication *in vitro*. Corroborating these findings, silencing autophagy in HeLa cells dampened PV replication (Jackson et al., 2005).

Arboviruses such as Dengue virus (DENV) and Zika virus (ZIKV), ssRNA+, have also been shown to induce the formation of membranes decorated with LC3 (Samsa et al., 2009; Liang et al., 2016). The mechanism relying upon autophagy-dependent DENV replication seems to involve the use of fatty acids generated during lypophagy, a specialized form of autophagy in which lipid droplets (LDs) are broken down for use in mitochondrial metabolism. DENV is known to increase LDs, which harbor viral capsid proteins. Thus, LDs provide a platform for viral replication (Samsa et al., 2009), a feature that also seems to occur in the case of SARS-CoV 2 (Dias et al., 2020). The pro-viral role of autophagy is elegantly illustrated in a study analyzing ZIKV vertical transmission. *Atg16l1*-deficient mice infected with ZIKV showed limited vertical transmission and placental and fetal damage (Cao et al., 2017). Furthermore, ZIKV promotes autophagy and dampens host efforts to induce another specialized form of autophagy, reticulophagy (or ER-phagy). The ER is a source of membrane for the establishment of viral replication. During ZIKV infection, reticulophagy is enhanced to restrain viral maturation, mediated by a protein called FAM134B. ZIKV-encoded NS3 cleaves FAM134B to suppress the formation of ER and viral protein-enriched autophagosomes, suggesting that the cleavage of FAM134B serves to specifically suppress the reticulophagy pathway (Lennemann and Coyne, 2017; Bhaskara et al., 2019). Recent findings showed that ZIKV, DENV, and PV regulate different subsets of autophagy initiation components for efficient viral growth in a non-canonical way. In common, all three viruses utilize the lipid scavenger protein Atg9 and recruit LC3 directly to membranes, bypassing the need for Atg5-mediated lipidation (Abernathy et al., 2019).

The induction of autophagosome formation and its maturation arrest has been demonstrated for human parainfluenza virus type 3 (HPIV3), and Influenza A virus (IAV), both ssRNA- viruses. This feature is also observed for some coronaviruses, as we will discuss later. The proposed mechanism involves the binding of viral proteins, such as phosphoprotein (P) of HPVI3 and matrix protein 2 (M2) of IAV, to cellular regulators of autophagy. In the case of HPVI3, SNARE domains of Synaptosome Associated Protein 29 (SNAP29) is bound to P, while Atg6/Beclin-1 and UVRAG containing PI-3 kinase complex is bound to M2 in the case of IAV (Gannage et al., 2009; Ding et al., 2014).

Once again, it is important to highlight that autophagy-related proteins might possess autophagy-independent effects. For example, LC3 and SNAP29 have been described to promote viral replication independently of the autophagic process (Monastyrska et al., 2013; Sharma et al., 2014; Alirezaei et al., 2015; Sarkar et al., 2021). For instance, non-lipidated LC3 is essential for double-membrane vesicles (DMVs) formation and replication of the ssRNA+ viruses, equine arteritis virus (EAV) and Japanese encephalitis virus replication (JEV) (Monastyrska et al., 2013; Sharma et al., 2014; Sarkar et al., 2021). Interestingly, coxsackievirus B3 replication is dependent on LC3 and autophagy but can still occur if non-lipidated LC3 is present and autophagy is absent (Alirezaei et al., 2015), showing surprising plasticity in terms of LC3-dependent processes of viral replication.

Viruses also impair viral recognition through autophagy modulation. Data from the literature demonstrate that HPIV3 suppresses innate immune responses after enhancing mitophagy to dampen viral recognition by mitochondria-located sensors, which blunts the production of IFN-I. More specifically, the matrix protein (M) of HPIV3 interacts with mitochondrial translation factor Tu (EF-TU) and binds to LC3 to promote autophagosome formation and mitochondrial degradation in a Parkin-PINK1 independent manner (Ding et al., 2017). A similar strategy of autophagic trafficking remodeling is used by enterovirus 65, a ssRNA+ virus, to replicate and exit from the cell without being degraded after lysosome fusion (Corona et al., 2018).

## Autophagy and Coronaviruses Infection (CoVs)

Much of our current knowledge on the interaction between autophagy machinery and CoVs rely on studies using mouse hepatitis virus (MHV) as a model, possibly due to its ability to be used in BSL-2 facilities and to infect multiple cell types and host species (de Haan et al., 2005). MHV infection leads to the formation of DMVs, closely resembling autophagosomes. In contrast to a well-established role of autophagy during infection with several viruses, its implication in the replication and pathogenesis of CoVs is still under investigation.

Initial studies on the interaction of autophagy and CoVs using MHV as a model demonstrated conflicting results. Prentice et al. (2004) showed that Atg5 was required to support viral replication in embryonic stem (ES) cells. The authors evaluated the replication of MHV in ATG5-deficient ES cells and observed a significant decrease in the number of plaque-forming units (PFU) (Prentice et al., 2004). A subsequent study revealed that ATG5 was dispensable for MHV replication in bone marrow macrophages and primary mouse embryonic fibroblasts (Prentice et al., 2004). Apart from the different cell types in these two studies, it is not possible to rule out a non-canonical role for Atg5. The hypothesis of non-canonical roles of ATG proteins is supported by findings that non-lipidated LC3-I localized to MHV-induced DMVs. For a long time, the origin of membranes composing the DMVs remained obscure. Currently, several pieces of evidence suggest the ER as the source. Two non-structural proteins (nsps 3 and 4) which have been suggested to be part of the replication-transcription complex (RTC), were shown to be N-glycosylated.

Interestingly, when ectopically expressed, nsp4 locates to the ER and moves to DMV upon infection (Oostra et al., 2007). In addition, ultrastructural studies report that DMVs are interconnected through their outer membranes as part of the reticulovesicular network (Knoops et al., 2008). Corroborating the hypothesis of an autophagy-independent origin of DMVs, Reggiori et al. (2010) demonstrated that DMVs originate from ER vesicular export containing non-lipidated LC3 and short-lived chaperones ER Degradation Enhancing Alpha-Mannosidase Like Protein 1 (EDEM-1) and OS9. One aspect against the idea of an ER origin for DMV membranes is that fragmentation of the Golgi apparatus contributes to DMV formation, a feature in which autophagy-related proteins also participate (Cortese et al., 2020).

Some shared features can be observed among SARS-CoV, SARS-CoV-2, and MERS-CoV infections *in vitro* concerning autophagy regulation, as extensively reviewed elsewhere (Shojaei et al., 2020; Zhao et al., 2021). Autophagy seems to be induced by all SARS-CoV, MERS-CoV, and SARS-CoV-2, with a potential role in viral replication (Chen et al., 2014; Yang et al., 2014; Gordon et al., 2020; Gassen et al., 2021). Direct regulation of autophagy by viral proteins, such as ORF3a and nsp6, is associated with autophagy induction (Cottam et al., 2011; Gassen et al., 2019; Qu et al., 2021). At the same time, the last step of autophagy seems to be negatively regulated by viral proteins, causing an accumulation of autophagosomes due to impaired fusion with lysosomes, both *in vitro* and *in vivo*, in the case of lung samples from deceased SARS-CoV-2 patients (Gassen et al., 2019; Gassen et al., 2021; Qu et al., 2021; Zhang et al., 2021). Altogether, these reports suggest that these viruses manipulate autophagy at multiple levels to its benefit, and specific autophagy modulators must overcome viral regulation to allow virophagy. In addition, as discussed earlier, autophagy is both regulated and an essential player in inflammation and resolution. Interestingly, autophagy is involved in the reduction of IL-17 secretion and NLRP3-dependent signaling (Reed et al., 2015; Cotzomi-Ortega et al., 2020), both possibly involved in coronavirus pathogenesis, and, pointed out as important mechanisms that govern bats, animals known for their resilience to viruses, disease tolerance (Ahn et al., 2019; Pacha et al., 2020; Rodrigues et al., 2020). Furthermore, autophagy restrains PRRs activation through a negative feedback loop (Zhu et al., 2019). Thus, autophagy modulators are promising drugs to restrain coronavirus pathogenesis by their anti-inflammatory effects and reduction of the intracellular viral load (Laing et al., 2019).

## Autophagy Pathways: Different Targets for Modulation

Autophagy can be regulated at both transcriptional and post-translational levels, and several proteins are known to coordinate the process, opening several new roads for specific targets modulation along the autophagy pathway.

The primary transcription factors involved in autophagosome formation and vesicular transport are TFEB, cyclic AMP response element-binding protein (CREB), and Forkhead box proteins (FOXOs 1, 3, 4, and 6), which are positive regulators of genes involved in autophagosome and lysosome biogenesis, and the negative ones, farnesoid x receptor (FXR) and Zinc Finger Protein With KRAB And SCAN Domains 3 (ZKSCAN3) (Di Malta et al., 2019). TFEB is a member of the helix-loop-helix leucine zipper (bHLH-Zip) family of transcription factors. Generally, under nutrient availability, TFEB is localized in the cytoplasm in its phosphorylated form (Sardiello et al., 2009). TFEB phosphorylation is mediated by different kinases, such as mTORC1, extracellular-signal-regulated kinase 2 (ERK2), glycogen synthase kinase 3 beta (GSKB3), Akt, and protein kinase C beta (PKCβ) (Martina et al., 2012; Li et al., 2016). mTORC1, ERK2, GSKB3, and Akt are responsible for TFEB retention on the cytoplasm. In contrast, PKCβ is associated with TFEB stability, supporting its activation after nuclear translocation (Peña-Llopis et al., 2011; Martina et al., 2012; Settembre et al., 2012; Ferron et al., 2013; Li et al., 2016; Palmieri et al., 2017). This indicates that specific phosphorylation sites are crucial for TFEB proper regulation. ZKSCAN3 is also regulated by mTORC1, opposing TFEB transcriptional activity under nutrient availability (Chauhan et al., 2013). TFEB can also be targeted by different phosphatases, favoring nuclear translocation and autophagy induction. For example, protein phosphatase 2A and calcineurin are responsible for TFEB activation after oxidative stress and calcium signaling, respectively (Medina et al., 2015; Martina and Puertollano, 2018; Xu et al., 2020). Once activated, TFEB translocates to the nucleus, where it binds to specific regulatory sequences (coordinated lysosomal expression and regulation- CLEAR) in the promoter regions of several genes that code for autophagy and lysosomal proteins, such as Atg9B, Beclin-1, Atg5, p62, and lysosomal transmembrane proteins and hydrolases, driving their transcription (Xu et al., 2020). TFEB is the target of different drugs, such as resveratrol, digoxin and curcumin (Xu et al., 2020). These drugs are being used in clinical trials against many diseases, in which their efficacy is being evaluated for their pleiotropic effects, which includes but is not restricted to autophagy induction, as discussed later.

FOXO transcription factors are also translocated to the nucleus once activated in the cytoplasm, promoting autophagy and lysosome-related genes transcription (Mammucari et al., 2007; Zhao et al., 2007; Sanchez et al., 2012). FOXO activity is modulated by Akt, which phosphorylates and restricts FOXO cytoplasmic localization (Brunet et al., 1999). Thus, different pharmaceuticals that target Akt can also impact FOXO activity, such as MK-2206 and BAY1125976, among others (Song et al., 2019). FOXO activity can also be regulated by AMPK (Song et al., 2019). Furthermore, sirtuins can positively regulate FOXO transcriptional activity, and the activity of Atg5, 7, and 8 are associated with autophagy induction (Ng and Tang, 2013). Interestingly, some of these modulators like MK-2206 and BAY1125976, and suramin, have been tested in clinical trials against tumors with good safety assessments (NCT01307631; Schneeweiss et al., 2019; NCT01671332).

CREB interacts with the coactivator CREB regulated transcription coactivator 2 (CRTC2) to induce Atg7, ULK1, and TFEB coding genes transcription, among other autophagy-related genes (Di Malta et al., Seok et al., 2014). FXR inhibits CREB interaction with CRTC2, restraining autophagy induction under nutrient availability (Seok et al., 2014). Both FXR and CREB are targeted by pharmaceuticals in different diseases (Barco et al., 2003; Wang et al., 2018; Sapio et al., 2020), with distinct proposed mechanisms of action. Among them, the FXR agonist, obeticholic acid, has been tested in different clinical trials, for example, in alcoholic liver disease, in which FXR agonists are thought to decrease the P450 2E1 enzyme responsible for oxidative stress-mediated injury in hepatocytes (Ali et al., 2015). CREB inhibitors have also been tested in anti-cancer therapies, aiming at the effects of CREB on cell proliferation and tumorigenesis-associated genes (Sapio et al., 2020).

**Figure 2:**
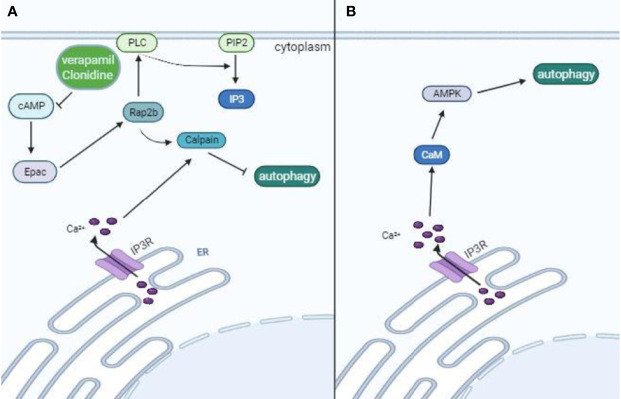
Opposing effects of intracellular Ca^+2^ in autophagy modulation. Calcium ions can inhibit or induce autophagy, depending on its levels and interactive proteins. Calcium release from the ER is mediated by IP3R, which links PLC activity to autophagy. IP3 is generated from PIP2 by PLC, which is dependent on free inositol levels. PLC activity also depends on cAMP levels that keep Epac and Rap2b activated. Rap2b-mediated activation of calpain drives Beclin-1 degradation, restraining phagophore formation. IP3R also recruits Beclin-1, which further restrains Beclin-1 availability from forming the class III PI3K complex. Thus, pharmaceuticals like verapamil and clonidine that lead to reduced cAMP levels are associated with autophagy induction **(A)**. On the other hand, high levels of intracellular Ca^+2^ lead to calmodulin activation and subsequent AMPK-induced autophagy after phosphorylation of ULK-1 complex and class III PI3K **(B)**.

At the post-translational level, different enzymes, kinases, and acetylases regulate protein complexes that coordinate the formation, elongation, and fusion of autophagosomes with lysosomes. As already discussed, the major complexes involved in the autophagic process are ULK1, ATG13, FIP200, and ATG101, which are responsible for the activation of the PI3K class III complex (Mercer et al., 2018), composed of Beclin-1, Vps15, Atg14L, and Vps34, which are responsible for autophagosome nucleation; two ubiquitin-like systems composed of the complex Atg12-Atg5-Atg16L and LC3 (Lahiri et al., 2019); and the complex that mediates lysosome fusion with autophagosomes, composed of Beclin-1, VPS34, VPS15 and UVRAG (Matsunaga et al., 2009). Several kinases and acetylases influence the activity of each complex. The major enzymes targeted by pharmacological modulators are AMPK, a positive regulator of Beclin-1 and ULK-1, activated by metformin (Nazarko and Zhong, 2013); mTORC1, a negative regulator of ULK-1 complex and TFEB, inhibited by rapamycin (Kim et al., 2011); and S-phase kinase-associated protein 2 (Skp2) and Akt that also affect different steps of the autophagic process. Akt can phosphorylate multiple targets, including mTORC1 and the transcription factor FOXO1, mediating autophagy inhibition (Dan et al., 2014; Pan et al., 2017). Skp2 is downstream from mTORC1 signaling, drives Beclin-1 degradation by ubiquitination (as an E3 ligase), and is also involved in epigenetic changes preventing TFEB transcriptional activity (Shin et al., 2016; Gassen et al., 2019). Many other players also affect autophagy, such as cyclic AMP (cAMP) and calcium. Calcium can induce or inhibit autophagy, depending on its levels and subcellular localization (Sun et al., 2016). In general, constitutive calcium release from the ER inhibits autophagy. It keeps mitochondrial tricarboxylic acid enzymes active and, consequently, high levels of ATP, which restrains AMPK activity (Ivanova et al., 2017). Calcium release from the ER can be mediated by the Inositol triphosphate (IP3) receptor (IP3R), linking phospholipase C (PLC) activity and autophagy (Putney and Tomita, 2012). PLC generates IP3 from phosphatidylinositol biphosphate (PIP2), and it is dependent on free inositol levels as an essential substrate for its enzymatic activity (Berridge, 2016). PLC activity is also dependent on cAMP levels that keep Exchange protein directly activated by cAMP (EPAC) activated and, consequently, Rap2b. Rap2b is a GTPase that interacts and activates PLC and calpain (Schmidt et al., 2001; Mestre et al., 2010). Calpain is a calcium-activated cysteine protease that inhibits autophagy through Beclin-1 degradation (Russo et al., 2011). Thus, PLC activity affects calcium release and is affected by cAMP. IP3R also directly inhibits the autophagic process through the recruitment of Beclin1, reducing its availability for the initiation complexes of autophagy (Vicencio et al., 2009). On the other hand, increased cytoplasmic calcium levels have also been associated with calmodulin activation (Keller et al., 2008), an upstream activator of AMPK and autophagy (Fogarty et al., 2010). Intriguingly, this process can also be activated by cAMP and EPAC1 (Laurent et al., 2015) (**Figure 2**). Altogether, as a complex and multiple-step process, autophagy can be manipulated in diverse ways, and the outcome will depend on the sum of positive and negative stimuli. Next, we will further discuss different pharmaceuticals that can modulate autophagy. Some of these drugs have already been approved for use in many different diseases with distinct proposed mechanisms of action, associated or not with autophagy modulation, and have been tested for coronavirus diseases. Thus, the United States Food and Drug Administration (FDA) approved the clinical use of statins, rapamycin, sirolimus, hydroxychloroquine, metformin, niclosamide, valinomycin, quercetin, digoxin, fluvoxamine, verapamil, clonidine, geldanamycin, loperamide, and miconazole in different diseases. Interestingly, the only drug approved by the FDA for treating viral infections (hepatitis B virus and hepatitis C virus) that is known to modulate autophagy (and many other anti-viral effects) is IFN-I. As already mentioned, IFN-I treatment has been tested in clinical trials for coronavirus diseases.

## Autophagy Modulators for Coronavirus Diseases Treatment

Different autophagy modulators are available or under investigation for their therapeutic effects in infectious and non-infectious diseases. Here, we discuss several studies that described the effects of these modulators in SARS-CoV, MERS-CoV, and SARS-CoV2 infections.

### Lysosome Alkalinizing Agents

Chloroquine and its analogs, like hydroxychloroquine, possess pleiotropic effects and have been used for years as antiparasitic and anti-inflammatory drugs to treat malaria and different inflammatory conditions, respectively. Chloroquine is known for its ability to inhibit lysosome acidic hydrolases, as a lysosomotropic alkalinizing agent. It also affects many processes within the cells and in mammalian physiologic and pathological events, for example inhibiting heme-mediated inflammation (Silva et al., 2021). Interestingly, chloroquine and hydroxychloroquine impaired *in vitro* viral replication by a not fully determined mechanism. It is believed that these pharmaceuticals drive a catastrophic accumulation of autophagosomes, leading to cell death and eliminating viral particles (Shojaei et al., 2020). However, *in vivo*, chloroquine reduces lung pathology in mice from SARS-CoV infection without affecting viral burden, suggesting an effect independent of viral replication (Barnard et al., 2006; Weston et al., 2020). Further studies are needed to confirm and better comprehend the impact of chloroquine on coronavirus infections.

### mTORC1 Inhibitors and AMPK Activators

Rapamycin and its analogs, inhibitors of mTORC1, are known inducers of autophagy affecting many different aspects of mammalian physiology, such as immune responses (Janes and Fruman, 2009). Rapamycin acts on cellular replication of SARS-CoV, MERS-CoV and SARS-CoV-2 in a specific manner, which correlates with the viral regulation of mTORC1 and related kinases. Similar to SARS-CoV-2, SARS-CoV infection seems to inhibit mTORC1, resulting in increased autophagic flux, contrary to MERS-CoV, which is associated with increased mTORC1 phosphorylation and kinase activity. Thus, rapamycin restrains viral replication in MERS-CoV infected hepatocytes (Kindrachuk et al., 2015) but increases viral replication in kidney cells infected with SARS-CoV-2 (Qu et al., 2021). In agreement with the effects of rapamycin in SARS-CoV-2 infection, AMPK activators, which also lead to autophagy assembly and initiation, were associated with increased viral replication (Gassen et al., 2021). Surprisingly, metformin (an AMPK activator)-treated patients seem to have a lower mortality rate (Bramante et al., 2021), but the role of autophagy in this supposed beneficial effect was not evaluated.

Sorafenib is a multikinase inhibitor that induces autophagy. It inhibits mTOR signaling and promotes the phosphorylation of AMPK, though this latter does not correlate with autophagy induction (Prieto-Domínguez et al., 2016). Sorafenib is approved for the treatment of hepatocellular carcinoma, as an inhibitor of growth factors signaling and, probably, as a regulator of autophagy as well. Interestingly, sorafenib has been described to inhibit both SARS-CoV-2 and MERS-CoV replications *in vitro* and is a compelling drug to be further evaluated in future clinical trials (Kindrachuk et al., 2015; Klann et al., 2020).

Recently, statins have been described as autophagy inducers (Zhang et al., 2013). The mechanism by which statins induce autophagy is not completed understood, but they do inhibit mTORC1 activity (Wei et al., 2013). Statins are currently used for the treatment of coronary heart diseases, with pleiotropic effects, including the improvement of endothelial function and the ability to lower low-density lipoprotein (LDL)-cholesterol levels. Interestingly, long-time users of statins seem to possess a lower risk for severe covid-19 development, despite increased ACE2 expression levels (Zhang X. J. et al., 2013). It is believed that the anti-inflammatory effects of statins are crucial for this protection, a feature that can be directly associated with autophagy induction (Peng et al., 2018).

Another interesting pharmaceutical that induces autophagy is ivermectin. It does so through regulation of mTOR/AMPK pathway (Liu et al., 2019; Zhang P. et al., 2020). Ivermectin is an antiparasitic drug with *in vitro* anti-viral properties, including against SARS-CoV-2 (Mastrangelo et al., 2012; Caly et al., 2020). Besides its effects on autophagy, ivermectin affects different cellular and physiologic processes, and, *in silico* analysis indicated a possible effect on viral enzymes as well, such as RNA-dependent RNA polymerase and 3-chymotrypsin like protease (3CL^pro^) (Heidary and Gharebaghi, 2020; Eweas et al., 2021; Mody et al., 2021). Thus, the mechanisms by which ivermectin inhibits viral replication (at least *in vitro*) are not fully established and its efficacy against covid-19 is being evaluated in distinct clinical trials with different results (Krolewiecki et al., 2021; Lim et al., 2022).

### Activators of Lysosomal Biogenesis

**Figure 3:**
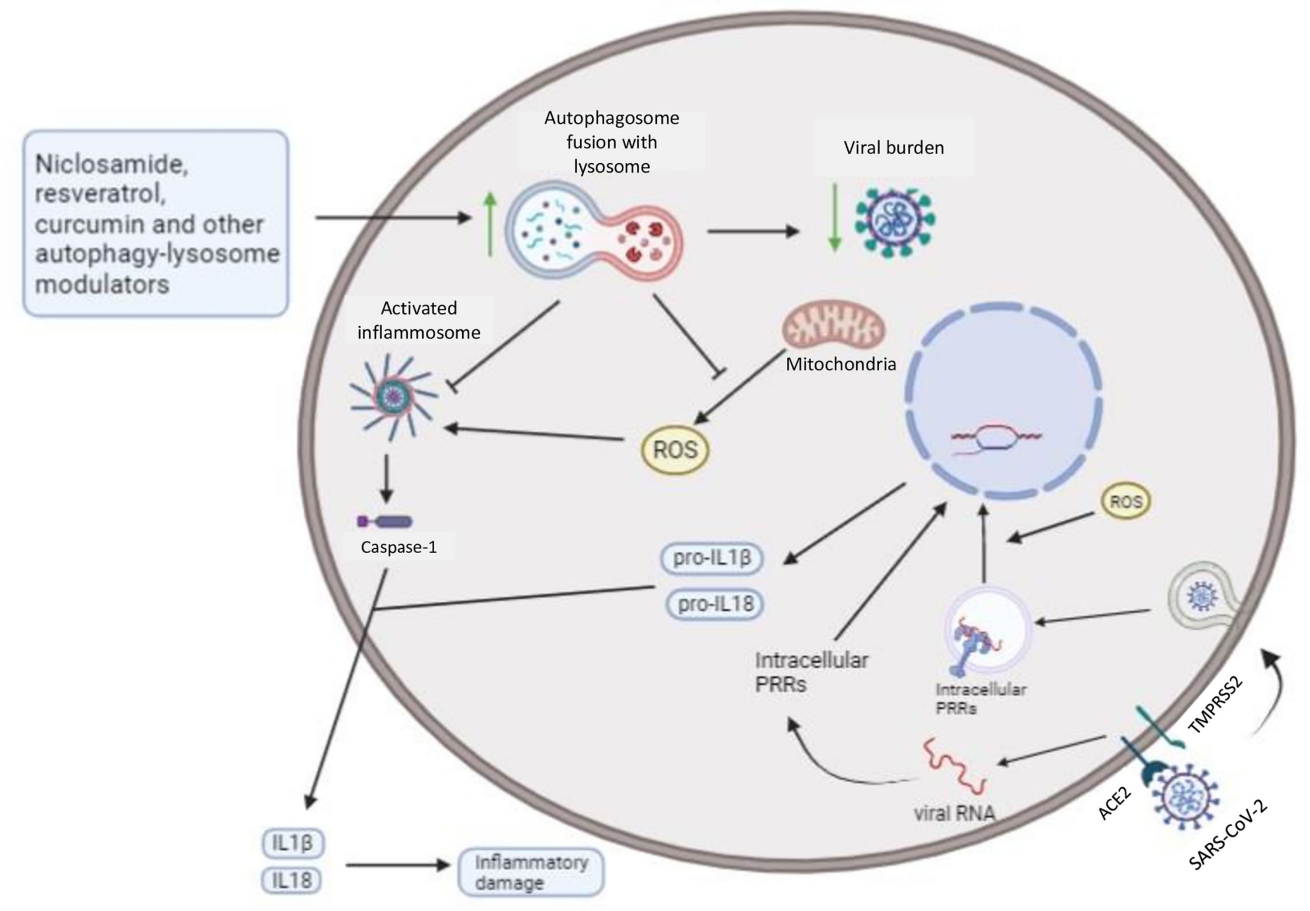
Autophagy can restrain intracellular viral burden and secretion of pro-inflammatory cytokines by infected host cells: Autophagy modulators that increase lysosome biogenesis and fusion with autophagosomes lead to degradation of viral particles, organelles, and macromolecules, like NLRP3, a known cytoplasmic PRR. Mitochondrial ROS also promotes both cytoplasmic, such as RLRs, and endosomal, such as TLR7 and TLR3, PRRs signaling, and mitophagy-mediated restriction of ROS can further restrain intracellular inflammatory pathways (from activated PRRs). Thus, niclosamide, resveratrol, and curcumin potentially drive increased resistance and disease tolerance, after autophagy induction, towards coronavirus diseases, reducing the intracellular viral burden and inflammatory mediators associated with tissue damage. Additionally, resveratrol and curcumin might also contribute to disease tolerance through its anti-oxidant activities, restraining mitochondrial ROS, a critical second messenger in PRRs signaling (Forman et al., 2010).

Modulators that target lysosomal biogenesis, like niclosamide and resveratrol, showed promising results *in vitro* regarding SARS-CoV-2 and MERS-CoV replication (Lin et al., 2017; Bramante et al., 2021). In addition, as previously discussed, autophagosome-lysosome fusion can restrain pro-inflammatory signaling and reduce inflammatory damage. Thus, several modulators that act on this step of autophagy and are known anti-inflammatory agents, such as valinomycin, curcumin, resveratrol, quercetin, digoxin, and niclosamide, are described as potential therapies against coronavirus diseases (Shen et al., 2019; Cho et al., 2020; Marinella, 2020; Pindiprolu and Pindiprolu, 2020; Zhang D. et al., 2020; Di Pierro et al., 2021; Ter Ellen et al., 2021; Thimmulappa et al., 2021). Niclosamide and quercetin have already been tested in clinical trials that confirmed both niclosamide safety, warranting further tests, and quercetin positive effects in patients, preventing severity (Backer et al., 2021; Di Pierro et al., 2021) (**Figure 3**). It is important to mention that these autophagy modulators have pleiotropic effects and may act on multiple targets across host cells and coronaviruses (Gibellini et al., 2015; Pandey et al., 2020). For example, valinomycin can also affect potassium equilibrium inside host cells, affecting viral replication (Sandler et al., 2020); quercetin, curcumin, and resveratrol showed *in silico* ability to bind S-ACE2 receptor complex, involved in SARS-CoV-2 entry, possibly inhibiting viral invasion (Pandey et al., 2020). Besides that, these compounds also affect mitochondrial metabolism and intracellular ROS generation (Ungvari et al., 2011; Sandler et al., 2020); niclosamide can affect SARS-CoV-2 entry as well, targeting the viral endocytosis process (Pindiprolu and Pindiprolu, 2020); and digoxin might impact on viral RNA synthesis through ion homeostasis misbalance (Cho et al., 2020).

### Suramin: A Sirtuin Activator

Suramin is an antiparasitic drug used to treat African sleeping sickness (Wiedemar et al., 2020). Suramin acts as an inducer of autophagy by deacetylating regulatory proteins (Trapp et al., 2007) and has been described to inhibit SARS-CoV-2 infection *in vitro* (Salgado-Benvindo et al., 2020). Thus, suramin repurposing to treat coronavirus diseases might be an interesting strategy to pursue. It seems that suramin modulates the early steps of viral replication cycle, probably viral binding and entry into the host cells (Salgado-Benvindo et al., 2020), and the exact role of autophagy modulation for the anti-viral effects of suramin were not established, but instigates further studies.

### Cyclic AMP and Calcium Modulators Driving Autophagy

Verapamil and clonidine are used as antihypertensive drugs, with distinct mechanisms of action. Clonidine acts as an agonist of the presynaptic alpha_2A_ receptor, depending on the autonomic nervous system for its effects (Szabo, 2002). Verapamil inhibits L-type calcium channels leading to vasodilation and relaxation of vascular smooth muscle cells, in an autonomic nervous system independent manner (Lee and Tsien, 1983). Clonidine inhibits cyclic AMP, while verapamil modulates intracellular calcium levels, leading to autophagy induction (Williams et al., 2008). A limited study made with only three patients showed a possible benefit associated with clonidine treatment (Hyoju et al., 2021). Though the mechanisms were not determined, the authors believe that sympathetic norepinephrine interference might reduce the deleterious inflammation associated with covid-19 (Hyoju et al., 2020). Verapamil is also believed to be an interesting pharmaceutical for the treatment of coronavirus diseases, since calcium entry during the initial life cycles of several viruses, including MERS-CoV, are very important (Straus et al., 2020). A clinical trial to determine the effects of verapamil treatment in covid19 was initiated, but suspended due to limited funding and number of patients (NCT04330300).

### Cellular Stressors as Inducers of Autophagy

Plumbagin and tunicamycin are stressors that induce autophagy as a response to ROS and ER stresses, respectively (Kuo et al., 2006; Ogata et al., 2006). Though both have been described as promising pharmaceuticals for the treatment of coronavirus diseases, they possess important toxicity, and, clinical trials associated with new formulations are needed. Tunicamycin inhibits crucial glycoproteins E2, S and M involved in SARS-CoV-2 replication and assembly (Dawood and Alnori, 2020), while plumbagin influences ROS levels, also supposedly impacting on viral replication (Nadhan et al., 2021).

### Autophagy activators with poorly determined mechanisms of action

The mechanisms by which some pharmaceuticals, like fluvoxamine, geldanamycin, miconazole, loperamide and rottlerin, activate autophagy are not completely understood (Cho et al., 2016; Qing et al., 2006; Qi et al., 2016; Tong et al., 2020; Ho et al., 2021). These drugs are used to treat many different diseases. Interestingly, they have been pointed out as exciting pharmaceuticals for the treatment of coronavirus diseases (Dawood and Alnori, 2020; Jayaseelan and Paramasivam, 2020; Karunakaran and Ganapathiraju, 2020; Klann et al., 2020; Lenze et al., 2020; Zhang D. et al., 2020; Lubkowska et al., 2021; Sanders et al., 2021). Once more, many of the mechanisms proposed for the beneficial effects of these pharmaceuticals were not related to autophagy modulation, as discussed next. Thus, the exact role of autophagy in the inhibitory activity of these pharmaceuticals still needs further investigation.

Fluvoxamine, a diffused antidepressant, was tested in a clinical trial to treat Covid-19 patients with positive effects, lowering the likelihood of clinical deterioration (Lenze et al., 2020). The proposed mechanism of the beneficial effects of fluvoxamine was the interference with serotonin-mediated thrombogenesis (Sukhatme et al., 2021).

Geldanamycin, an inhibitor of the chaperone Hsp90, presents promising results for cancer therapy in pre-clinical tests (Ochel et al., 2001). Though geldanamycin toxicity and instability precluded clinical trials, its analogs are showing important improvements concerning deleterious side-effects (Dey and Cederbaum, 2006; Kim et al., 2009). Several studies showed an important role of Hsp90 in different virus replication, including coronaviruses (Li et al., 2004; Li et al., 2020; Lubkowska et al., 2021). Hsp90 is crucial for appropriate viral proteins folding, making it a compelling molecular target (Li et al., 2020). Thus, the inhibitory effects of geldanamycin in MERS-CoV and SARS-CoV-2 replication *in vitro* were recently evaluated and proved (Li et al., 2020). In addition, geldanamycin also possesses anti-inflammatory effects (Kasperkiewicz, 2021), placing it as an important alternative for coronavirus diseases treatment.

Miconazole is used to treat fungal infections (Heel et al., 1980) and has been recently described, *in silico*, for its ability to bind and probably inhibit SARS-CoV-2 main protease CL^pro^, also called M^pro^ (Ferraz et al., 2020). Further studies are necessary not only to confirm a possible inhibitory effect of miconazole during viral replication but also to establish if autophagy induction contributes to this inhibition. In addition, other viral M^pro^ inhibitor, nelfinavir, which restrains SARS-CoV-2 replication *in vitro*, is also able to induce autophagy (Gills et al., 2008).

Loperamide is used as an agonist of opiate receptors reducing intestinal motility. As discussed earlier, coronavirus diseases might induce gastrointestinal symptoms and loperamide can be used to treat diarrhea. Unexpectedly, loperamide showed inhibitory activity *in vitro* for MERS-CoV and SARS-CoV-2 infection. The authors proposed that loperamide inhibition of 3C^pro^ and papain-like protease (PL^pro^) was responsible for the viral replication impairment (Kuo et al., 2021).

At last, rottlerin acts as an inhibitor of different kinases (like PKC and calcium modulator kinase III) that affect multiple cellular processes, including autophagy (Williams et al., 2008; Xiang et al., 2020). Thus, rottlerin possesses pleiotropic effects, suppressing *de novo* lipogenesis in adipocytes and tumor growth *in vitro* (Ma et al., 2018; Kim and Go, 2021). Computational analysis indicated rottlerin as a promising drug that targets SARS-CoV-2 protease M^pro^ (Glaab et al., 2021). However, rottlerin inhibited the anti-MHV-1 activity of IFNα (Zorzitto et al., 2006), and many more studies are required to validate this drug for use in covid-19 patients.

## Discussion

We reinforce that autophagy modulators are exciting candidates for drug-repurposing to treat coronavirus diseases, mainly if used in combination with other treatment modalities. Multifactorial diseases, such as viruses, usually require combinatorial therapies in order to be effective. Thereby, pharmaceuticals that block coronavirus replication, such as the ribonucleoside analog MK4482 (Rosenke et al., 2021), can be combined with several auto-lysosome modulators, like curcumin and resveratrol, restraining both viral replication and increasing viral macromolecules degradation by xenophagy. Furthermore, inhibitors of viral proteases that are crucial for viral proteins maturation after polyproteins catalysis, such as PF-07321332 (clinical test NCT04960202), can also be combined with auto-lysosome activators. Another promising strategy is to combine autophagy modulators with IFNs administration. As discussed earlier, it has been described that autophagy is induced by IFNs, a feature that can contribute to the antimicrobial effects of IFNs (Tian et al., 2019). Thus, the combination of IFNs and autophagy modulators would allow an additive effect, increasing host resistance while providing an essential control to inflammatory damage, as autophagy can also exert anti-inflammatory effects. During the late inflammatory phase of SARS-CoV-2 disease, characterized by immune dysfunction and increased inflammatory damage, a combination of antibiotics, like azithromycin, corticosteroids, and autolysosome activators, can protect against secondary bacterial infections and inflammatory damage. Autolysosome modulators and antibiotics can restrain bacterial infections through increased resistance due to induction of xenophagy and bacterial metabolism blockage, depending on the type of antibiotics used. In addition, autolysosome inducers are possible regulators of inflammatory mediated damage through PRRs signaling and inflammasome restriction, also contributing to corticosteroid control of inflammation-mediated tissue damage.

## Concluding Remarks

Autophagy is a critical stress response mechanism that has a back-and-forth interplay with the immune system and can impact pathological processes associated with inflammation. In addition, many viruses can manipulate autophagy machinery to support viral replication or prevent viral components degradation by lysosomes. Different studies support the idea that coronavirus modulates the autophagic machinery positively while inhibiting autophagosome fusion with lysosomes. Thus, autophagy modulation is an attractive therapeutic strategy to increase disease tolerance and resistance to coronavirus infections. The central purpose of these modulators is to increase autophagosome fusion with lysosomes, allowing proper virophagy, while restraining NLRP3-dependent cytokines (IL-18 and IL-1β) release, mitochondrial ROS-PRRs signaling pathways, and promoting the anti-inflammatory effects of lipid mediators, leading to increased disease tolerance. Thus, these autophagy modulators would reduce pathological inflammation and viral replication within the cells. Interestingly, several pharmaceuticals under clinical tests for coronavirus diseases have shared the ability to induce autophagy. These compelling findings might not be fortuitous but indicate that autophagy can contribute to or even be the central mechanism behind the supposed efficacy of certain pharmaceuticals.

## Author Contributions

RS contributed with text writing and figures elaboration; JR contributed with figures elaboration and text review and edition; GdS contributed with text review, edition and elaboration; LdC contributed with text review, edition and elaboration; LT contributed with text writing, edition and review, and figure elaboration. All authors contributed to the article and approved the submitted version.

## Funding

The fundings here listed supported the researchers payment: Fundação Carlos Chagas Filho de Amparo à Pesquisa do Estado do Rio de Janeiro (FAPERJ-Rio de Janeiro- Brazil); Coordenação de Aperfeiçoamento de Pessoal de Nível Superior (CAPES-Brazil) Epidemias AUXPE: 666/2020; Conselho Nacional de Desenvolvimento Científico e Tenológico (CNPq- Brazil).

## Conflict of Interest

The authors declare that the research was conducted in the absence of any commercial or financial relationships that could be construed as a potential conflict of interest.

## Publisher’s Note

All claims expressed in this article are solely those of the authors and do not necessarily represent those of their affiliated organizations, or those of the publisher, the editors and the reviewers. Any product that may be evaluated in this article, or claim that may be made by its manufacturer, is not guaranteed or endorsed by the publisher.
